# Metabolite Profiles Provide Insights into Underlying Mechanism in *Bupleurum* (Apiaceae) in Response to Three Levels of Phosphorus Fertilization

**DOI:** 10.3390/plants11060752

**Published:** 2022-03-11

**Authors:** Jialin Sun, Zejia Duan, Ye Zhang, Sisi Cao, Zhonghua Tang, Ann Abozeid

**Affiliations:** 1 College of Chemistry, Chemical Engineering and Resource Utilization, Northeast Forestry University, Harbin 150040, China; klp15sjl@nefu.edu.cn (J.S.); duanzejia@nefu.edu.cn (Z.D.); yzhang@nefu.edu.cn (Y.Z.); 2 Biological Science and Technology Department, Heilongjiang Vocational College for Nationalities, Harbin 150066, China; 3 Medical Department, Harbin Vocational & Technical College, Harbin 150040, China; shilei@hljucm.net; 4 Botany and Microbiology Department, Faculty of Science, Menoufia University, Shebin Elkoom 32511, Egypt

**Keywords:** *Bupleurum*, phosphorus nutrition, primary metabolism, metabolite profiles

## Abstract

Phosphorus (P) deficiency affects plant yield and quality, yet at the same time, excessive phosphorus application does not necessarily promote the growth of plants. How to maintain a balance between biomass accumulation and phosphorus application is a problem. Therefore, the purpose of this research was to explore the relationship between yield and quality of *Bupleurum* and phosphorus fertilization, based on three phosphorus fertilization levels (20 kg∙ha^−1^; 10 kg∙ha^−1^; and 0 kg∙ha^−1^). We adopted gas chromatography-mass spectrometry to assess the response of primary metabolites of different plant tissues (flowers, main shoots, lateral shoots and roots) to phosphorus fertilization. At the same time, high-performance liquid chromatography was used to quantify saikosaponin A and saikosaponin D, the main active ingredients of *Bupleurum*. Our research showed that low phosphorus level application has a positive impact on the yield and quality of *Bupleurum*, especially the above-ground parts increasing the fresh weight of flowers and lateral shoots and the length of main shoots, and moreover, increasing the saikosaponins content in all above-ground parts while decreasing the content in roots which show no significance increase in fresh weight and length. However, high phosphorus level showed a negative impact as it decreases the saikosaponins content significantly in flowers and roots. Furthermore, phosphorus application changed the proportion of saikosaponins, promoting the content of saikosaponin A and inhibiting the content of saikosaponin D in most organs of *Bupleurum*. Therefore, we can say that high phosphorus application is not preferable to the yield and quality of *Bupleurum.* To identify the metabolic pathways and special key metabolites, a total of 73 metabolites were discovered, and four differential metabolites—ether, glycerol, chlorogenic and L-rhamnose—were considered to be the key metabolites of *Bupleurum*’s response to phosphorus fertilization. Furthermore, *Bupleurum*’s response to phosphorus fertilization was mainly related to metabolic pathways, such as starch and sucrose metabolism and galactose metabolism. Under the phosphorus level, the content of sugars, organic acids and their derivatives, polyols and their derivatives and alkyl were upregulated in flowers. Furthermore, the contents of compounds in the main shoot and lateral shoots showed the same upward trend, except glycosides and polyols and their derivatives.

## 1. Introduction

As it is known, the compositions and contents of the plant largely depend on genetics, growth stage, environment and cultivation, and so on [1]. Among numerous factors, the availability of plant nutrients has received more attention. Phosphorus (P) is an imperative nutrition element for plant growth and development, which affects energy metabolism, nucleic acid synthesis, glycolysis in plants and vital physiological processes [2,3,4]. As the available soil phosphorus is low, crop yield and growth is limited [5]. In order to cope with phosphorus deficiency, plants have developed flexible strategies through complex biological processes, such as changing metabolic processes, regulating root system structure, accumulating and secreting organic acids, making plant roots and mycorrhizas fungal symbiosis [6,7,8]. Changes in root system structure in reaction to insufficient phosphorus are usually involved in phosphorus uptake, while changes of metabolic processes are related to phosphorus use efficiency [9]. In addition, rational phosphorus fertilization and improved phosphorus use efficiency are effective methods to ensure sustainable development of agricultural production [10,11].

Chinese medicinal materials, as a typical complex chemical system, have multi-component, multi-target and synergistic effect characteristics. It is indispensable to comprehensively evaluate the quality of the chemical components of Chinese herbal medicines. Metabolomics has opened up a new way to qualitatively and quantitatively analyze all low-molecular-weight metabolites of a given organism or cell over a specific physiological period, and detect changes in metabolites in response to endogenous and exogenous stimuli [12,13,14]. It not only has the value of prediction, diagnosis and drug application in disease research, but has also been widely used in the characterization of natural products, evaluation of therapeutic effects and analysis of potential mechanisms [15]. During the last years, this technique has been successfully applied to focus on the mechanism of secondary metabolic pathways, such as identifying the potential biomarkers to control product quality and contributing to improve disease resistance [16,17]. In addition, it has been widely used in research on active components and quality evaluation of traditional Chinese medicine, which could help to obtain a complete picture of the metabolic reactions of medicinal plants [18,19]. Meantime, metabolomics analysis has been used to reveal the mechanism of low nutrient tolerance in plants, so as to supply a foundation for the effective development of plant quality control and germplasm improvement. The typical examples include the study of the effect of nitrogen and phosphorus fertilization on metabolome of goji fruit [20,21]. The purpose of metabolomics comprehensively analyze all metabolites in biological samples, and demonstrate huge potential in clarifying the metabolic processes of plants [22]. A few metabolomics studies have been conducted in medicinal plants, such as *Panax ginseng* [23] and *Rehmannia glutinosa* [24], as well as *Astragalus mongholicus* [25]. In contrast, the metabolomic profile of *Bupleurum* has not been investigated extensively.

*Bupleurum* is a perennial herb belonging to the family Apiaceae, with about 200 species all over the world [26]. It is grown diffusely worldwide, including in temperate zones of the Northern Hemisphere, Europe, North Africa, North America and Asia [27]. Bupleuri Radix, the root of *Bupleurum scorzonerifolium* Willd. or *Bupleurum chinense* DC., has a long history of use and important economic value in China [28,29]. Due to its remarkable and stable clinical efficacy, it is included in the 2020 Chinese Pharmacopoeia [30]. Active compounds isolated from the roots, such as volatile oils, flavonoids, saikosaponins and polysaccharides [31,32], possess various pharmacological functions, including common cough, fever and influenza, hepatitis, malaria menoxenia, thoracolumbar pain, and uterine as well as rectal prolapse [33,34,35,36]. Studies have shown that roots contain saikosaponin A, C and D, but saikosaponin A and D are the main active ingredients [37].

Recently, gas chromatography (GC) or liquid (LC) chromatography combined use with mass spectrometry were major technical methods for metabonomic analysis [38]. Due to sensitive selective mass detection, high separation ability and reproducible retention times, gas chromatography-mass spectrometry (GC-MS) is a very suitable technique for metabolomics analysis [39,40]. GC-MS could be used to determine a variety of primary metabolites, such as carbohydrates, organic acids, fatty acids, amino acids and so on [41]. Liquid chromatography-mass spectrometry (LC-MS) could be used to analyze secondary metabolites, such as phenylpropanoids, lignins, terpenes and phenolic compounds [42]. The combined analysis of GC-MS and LC-MS could significantly increase the range of metabolites, and then comprehensively summarized and analyzed the changes of metabolites in detail [43].

Despite many studies have confirmed biological activities and pharmacological properties of *Bupleurum*, the relationship between the metabolome and phosphorus fertilization of *Bupleurum* is still unclear. At this study, we applied gas chromatography-mass spectrometry to compare the metabolite profiles of different tissues of *Bupleurum* (roots, main shoots, lateral shoots, and flowers) under different levels of phosphorus fertilization. Our aim was to investigate the following: (1) effect of phosphorus fertilization on the mass accumulation and partition in *Bupleurum*; (2) changes in the metabolite profiles of different tissue under different level of phosphorus fertilization; and (3) key metabolites associated with phosphorus fertilization to explain key points of biosynthesis regulation. Therefore, studying overall metabolic changes of *Bupleurum* under phosphorus fertilization will provide a basis for nutritional regulation.

## 2. Results

### 2.1. Comparative Analysis of Quality and Traits of Bupleurum under Different Phosphorus Level

**Figure 1:**
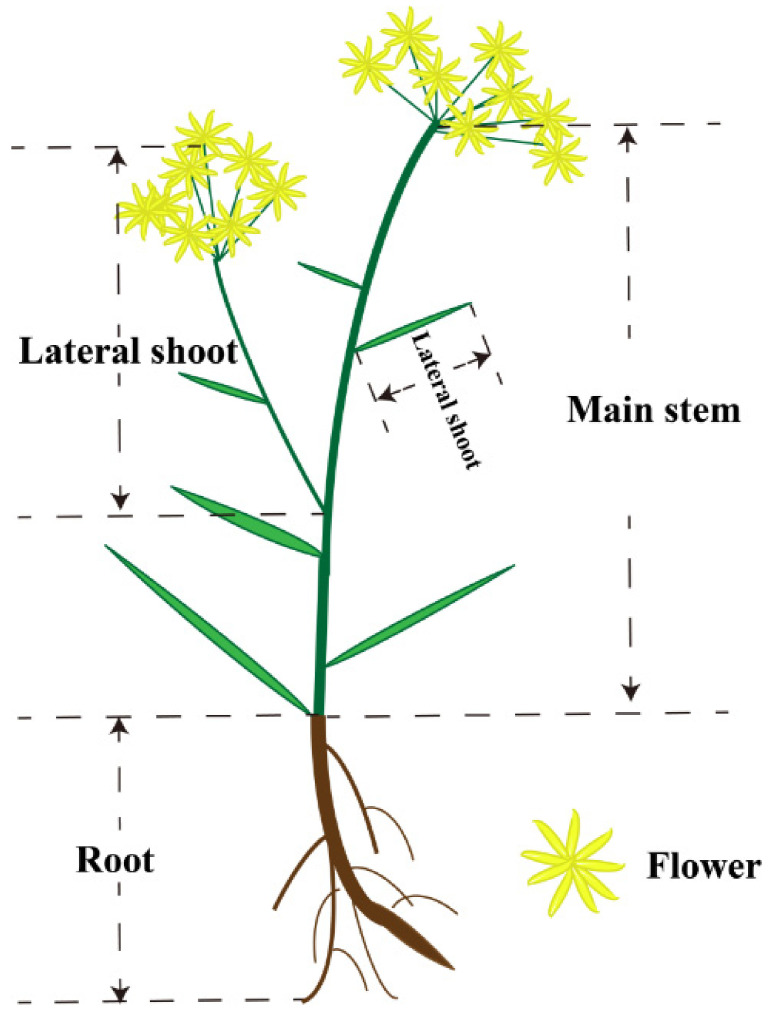
The whole *Bupleurum* was divided into four organs, which are the root, main shoot, lateral shoot and flower.

**Figure 2:**
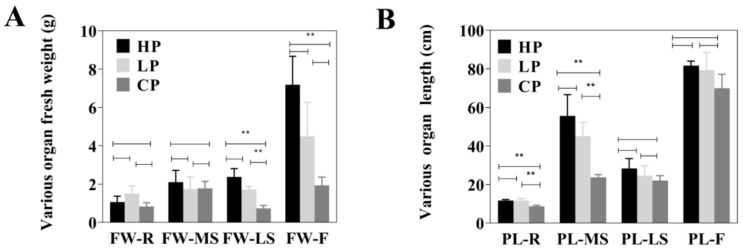
Growth performances of three concentrations of phosphorus fertilization. (**A**) Various organs fresh weight (*n* = 3); (**B**) Various organs length (*n* = 3). Three phosphorus fertilization levels: low phosphorus (LP), control group (CP) and high phosphorus (HP); and Four organs: root (R), main shoot (MS), lateral shoot (LS) and flower (F). FW: fresh weight; PL: plant organ length. ** represent *p* < 0.01.

In this experiment, the whole *Bupleurum* individuals growing under three levels of phosphorus fertilization—low-phosphorus (LP), control-phosphorus (CP) and high-phosphorus (HP)—were divided into four parts (Figure 1), including flowers (F), main shoots (MS), lateral shoots (LS) and roots (R). We determined the fresh weight and length for the four parts to find the influence brought by different phosphorus fertilization levels. The result revealed that different levels of phosphorus fertilization showed a significant effect on the yield and biomass of *Bupleurum* (Figure 2). In details, the fresh weight of *Bupleurum*’s lateral shoots and flowers significantly increased (*p* ≤ 0.01) under LP level compared with the CP level, and a further weight increase was observed under HP level (Figure 2A). However, no significant increase in fresh weigh of main stem and roots was observed with phosphorus application. Considering the organs’ length (Figure 2B), the situation was reversed, and the increase in the length of lateral shoots and flowers was not significant (*p* ≥ 0.05), while a significant increase in the main stems length was observed under phosphorus fertilization.

### 2.2. Total Saikosaponins Content Accumulated in Bupleurum Different Tissues under Three Levels of Phosphorus Fertilization

**Figure 3:**
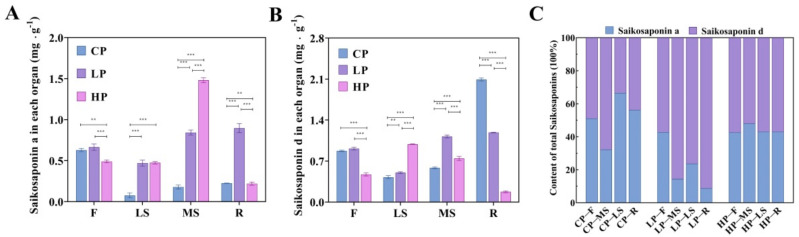
(**A**) Saikosaponin A analysis at three phosphorus fertilization levels and four organs. (**B**) Saikosaponin D analysis at three phosphorus fertilization levels and four organs. (**C**) The percentage of saikosaponins content, including saikosaponin A and D. Three phosphorus fertilization levels: low phosphorus (LP), control phosphorus (CP) and high phosphorus (HP); and Four organs: flower (F), main shoot (MS), lateral shoot (LS) and root (R). ** represent *p* < 0.01; *** represent *p* < 0.001.

Initially, this study exploited a HPLC method for simultaneous quantification of saikosaponin A and saikosaponin D. Simultaneously, we also measured the total content of saikosaponins in the different *Bupleurum* tissues under the three levels of phosphorus fertilization. We found that under control phosphorus level, the total saikosaponin content was the highest within roots, phosphorus application decreased the content in roots, while it increased in the above-ground organs. The decrease in roots’ total saikosaponin was very significant under a high phosphorus level. Moreover, comparing with low phosphorus level, high phosphorus shows an increasing in saikosaponin content in only the main stem and lateral stem, while the saikosaponins in flowers and roots decreased significantly, especially in roots (Figure 3A,B). When the total saikosaponins content was fixed and we compared among Saikosaponin A and Saikosaponin D, we found that when there was no phosphorus, and the Saikosaponin D percentage was higher than the percentage of saikosaponins A in all organs. With the increase of phosphorus fertilizer content, the proportion of Saikosaponin D gradually decreased, and Saikosaponins A increased instead (Figure 3C).

### 2.3. Overview of the Metabolites Profiles in Response to Three Levels of Phosphorus Fertilization

**Figure 4:**
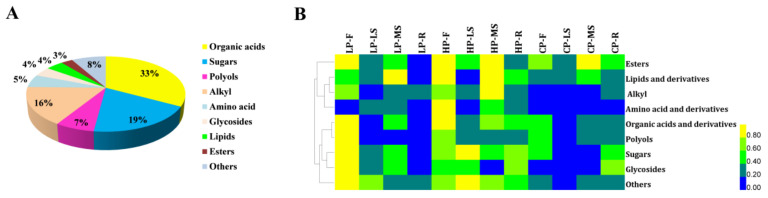
(**A**) Classification of 73 metabolites of *Bupleurum*. (**B**) The cluster analysis and heat map for *Bupleurum* plant parts under different levels of phosphorus fertilization. CP: control phosphorus; LP: low phosphorus; HP: high phosphorus; R: root; MS: main shoots; LS: lateral shoots; F: flower. The down-regulated and up-regulated and metabolites are represented as blue and yellow, respectively. The blue color means that abundance is 0, as shown in the lower right corner.

In order to understand more clearly the changes of metabolites in different phosphorus levels, GC-MS was used to the samples under phosphorus stress. A total of 73 primary metabolites were identified, which could be further divided into 9 groups, including 24 organic acids, 14 sugars, 12 alkyls, 5 polyols, 4 amino acids, 3 lipids, 3 glycosides, 2 esters and 6 others (Figure 4A). The clustering heat map was performed to assess the differences among the *Bupleurum* metabolites profiles in different tissues under three phosphorus levels. Two main clusters were obtained according to the relative differences of *Bupleurum* accumulation patterns (Figure 4B). The results indicates that different tissues have similar metabolic patterns. For example, with phosphorus application, metabolites such as esters, lipids, organic acids, sugar and glycosides accumulate preferentially within flowers and main shoots, while their overall levels within roots and lateral shoots are relatively low. However, roots and lateral shoots have substantially higher levels of sugar and glycosides under high phosphorus levels (Figure 4B). These specific metabolites likely reflect that the accumulation of metabolites during *Bupleurum* development is tissue-specific.

**Figure 5:**
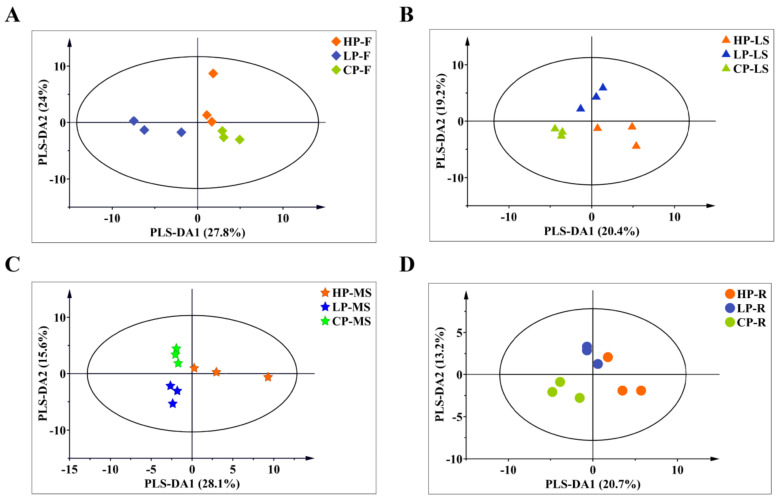
Score plots of PLS−DA for metabolites variation in flowers (**A**), lateral shoots (**B**), main shoots (**C**) and roots (**D**). Different markers and colors represent different meanings; ●, ◆, ▲, ★ represent roots (R), flowers (F), lateral shoots (LS), main shoots (MS), respectively; and red, green and blue represent high phosphorus (HP), low phosphorus (LP) and control phosphorus (CP), respectively.

The PLS-DA could evaluate discrepancy between various phosphorus levels of multiple variables through some principal components (Figure 5). Obviously, the PLS-DA results showed that the samples of different phosphorus levels were clearly separated in all tissues, which indicated that the metabolic differences were significant, corresponding to the results of cluster analysis and heat map. The first partial least-square method (PLS-DA1) could account for approximately 27.8%, 20.4%, 28.1% and 20.7% of the variation in four tissues (flower, lateral shoot, main shoot, root) separately. In terms of composition, it can be found that for the flowers and main shoots, HP, LP and CP are completely separated. However, the lateral shoot and root showed that CP and LP groups were significantly separated. The second partial least-square method (PLS-DA2) could explain 24%, 19.2%, 15.6% and 13.2% of the features of the original data set.

### 2.4. Metabolite Profiling of Bupleurum under Phosphorus Fertilization in KEGG Enrichment Analysis and Volcanic Map

The metabolic profiles of *Bupleurum* were analyzed using a fold change value ≤ 0.05 and a *p* value ≥ 1. In most cases, the active ingredients of *Bupleurum* were closely related to the level of phosphorus fertilization (Table 1). Comparing HP with CP, significant changes occurred in 22 metabolites, including 18 up-regulated metabolites (8 F, 5 LS, 4 MS, 1 R) and 4 down-regulated metabolites (1 LS, 1 MS, 2 R) in the root. About 26 metabolites had changes when comparing LP to CP, with 20 (12 F, 4 LS, 4 MS) down-regulated and 6 up-regulated metabolites (1 F, 1 LS, 4 MS). Compared with HP, 17 metabolites were significantly changed in the LP group, including 8 (2 F, 2 LS, 3 MS, 1 R) up-regulated and 9 (5 F, 2 LS, 2 MS) down-regulated metabolites.

**Figure 6:**
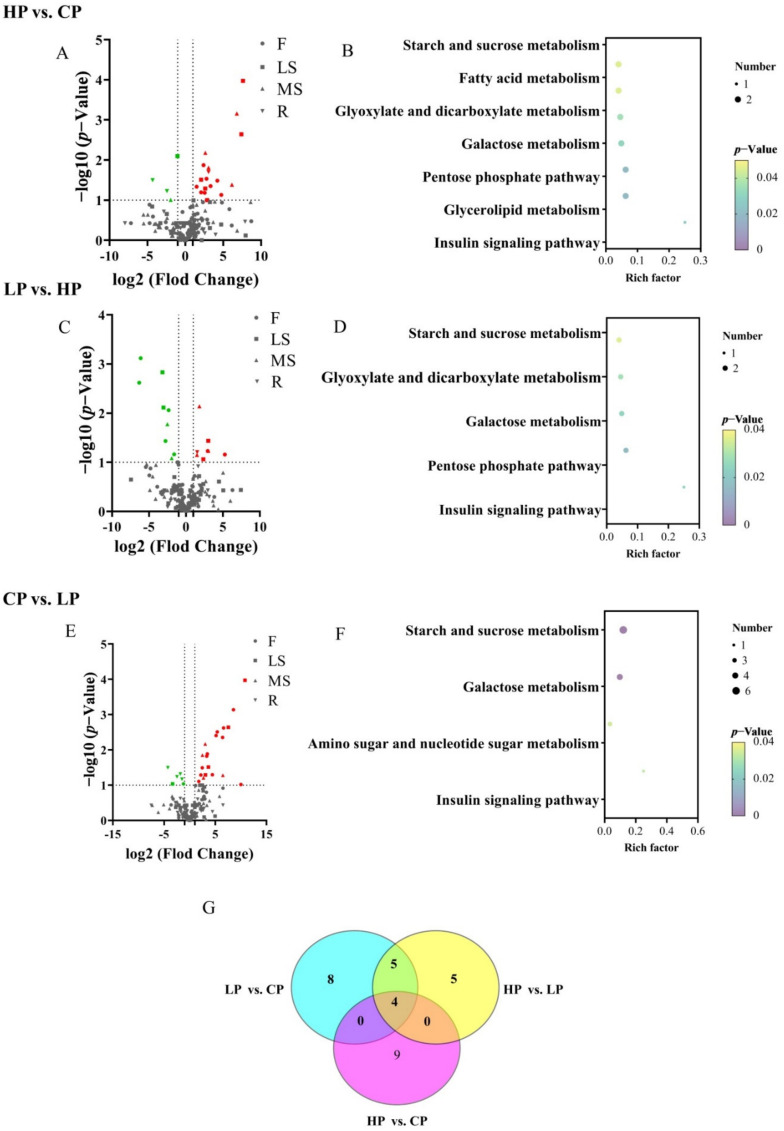
(**A**–**F**) The volcanic map of differential metabolites and enrichment analysis of KEGG. The red and green colors represent the content of metabolic that was up-regulated and down-regulated, respectively; ●, ▇, ▲, ▼ represent flowers, lateral shoots, main shoots and roots, respectively. The *p*-value indicates the degree of enrichment, and the closer the *p*-value is to 0, the more remarkable the enrichment. The point stand for the quantity of differential metabolites. (**G**) The Venn diagram of differential metabolites.

The Kyoto Encyclopedia of Genes and Genomes (KEGG) and enrichment results show that the effects of different levels of phosphorus fertilization (LP vs. CP, LP vs. HP, HP vs. CP) on metabolic pathways mainly involve the starch and sucrose metabolism and galactose metabolism (Figure 6). In HP vs. CP, various metabolites were mostly focused on glycerolipid metabolism, starch and sucrose metabolism, galactose metabolism, pentose phosphate pathway, fatty acid metabolism, glyoxylate and dicarboxylate metabolism, and even insulin signaling pathway (Figure 6 A,B). For HP vs. LP, the difference of KEGG enrichment classification was also related to the following pathways: starch and sucrose metabolism, glyoxylate and dicarboxylate metabolism, galactose metabolism, pentose phosphate pathway and insulin signaling pathway (Figure 6 C,D). For LP vs. CP, metabolic pathways of differential metabolites are involved with starch and sucrose metabolism, insulin signaling pathway, amino sugar and nucleotide sugar metabolism and galactose metabolism (Figure 6 E,F). The collective metabolic pathways are starch and sucrose metabolism and galactose metabolism, which are mainly related to sugars. The appraisal of potential metabolites involved into phosphorus stress might contribute to the growth and development of *Bupleurum*. We performed a Venn diagram (Figure 6G) to depict shared metabolites of different expressions among HP vs. CP, CP vs. LP and HP vs. CP. We found that the abundances of ether, glycerol, chlorogenic or L-rhamnose had an obvious change in the response to phosphorus fertilization; therefore, these four over-lapping metabolites could be regarded as key metabolites. Moreover, 9, 4 and 4 differential metabolites existed solely in (LP vs. CP) vs. (HP vs. LP), (LP vs. CP) vs. (HP vs. CP) and (HP vs. LP) vs. (HP vs. CP), respectively.

### 2.5. Metabolic Network Diagram and Potential Metabolites in Bupleurm under Three Levels of Phosphorus Fertilization

**Figure 7:**
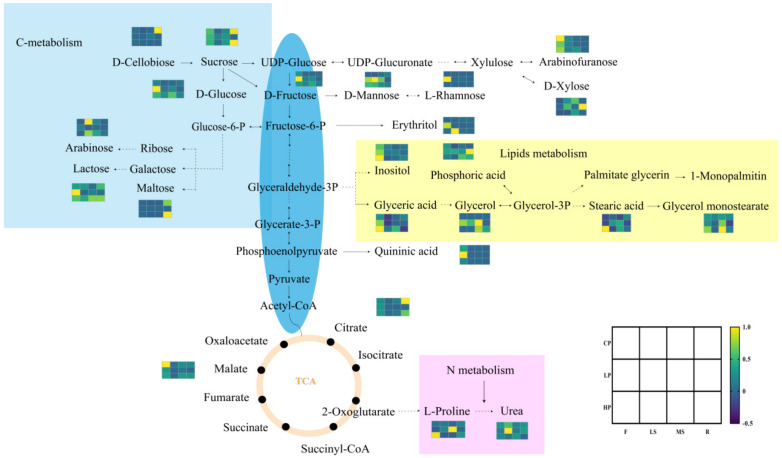
Visualization of primary metabolites in biochemical pathways, and the cluster analysis and heat map for metabolite classification in phosphorus stress.

On the basis of the KEGG annotation and enrichment data, significant metabolites were mapped to C-metabolism, lipids metabolism pathways to guarantee clear changes in the metabolic regulation of phosphorus (Figure 7). The overlap of differentially expressed metabolites was observed, suggesting the partial similarity of mechanisms of *Bupleurum* in response to phosphorus fertilization. The metabolic network diagram went on to verify the above hypothesis. Responses to phosphorus fertilization of *Bupleurum* were dynamic, and included intricate pathways. Therefore, phosphorus fertilization may activate some key physiological and metabolic activities that lead to the growth and development of *Bupleurum*.

## 3. Discussion

### 3.1. Influences of Phosphorus Fertilization on Bupleurum Yield, Quality and Saikosaponins Contents

Phosphorus deficiency severely restricts the growth and productivity of plants. Phosphorus can make stems and branches more rigid, cause early flowering and fruiting, and can improve the disease resistance and stress resistance of plants [44,45]. Plant Fresh weight and length are two important evaluation indexes of *Bupleurum* quality. Our results revealed that the fresh weight of flowers and lateral shoots showed significant increase under HP levels, compared with CP treatments. It is said that the long-term phosphorus deficiency could arrest proliferation of lateral shoots [46]. Moreover, similar results reported that high phosphorus levels increase flower number and weight [47,48,49]. The reason for this could be that the use of phosphorus fertilization encourages the accumulation of nutrients, such as sugars, organic acids and polyols. Plant roots play an important role in phosphorus absorption. In many studies, the rhizome ratio of phosphorus-stressed plants increased compared with phosphorus-sufficient plants due to the proliferation of lateral roots in phosphorus-stressed plants, in order to promote phosphorus absorption when phosphorus availability decreases [46,50,51,52]. This could explain our results, which show that low and high phosphorus application did not show a significant increase in root weight or length (Figure 2).

Recent studies indicated that saikosaponins A and D are the main active components to which the clinical efficacy evaluate the quality of *Bupleurum* in the Chinese Pharmacopoeia [37]. Therefore, we studied the relationship between phosphorus fertilizer application and the content of saikosaponins A and D in *Bupleurum*. Although phosphorus fertilization promoted the fullness of the shoots and leaves [53], high phosphorus levels (HP) have a limited or even negative impact on saikosaponins content within *Bupleurum*, decreasing the content in roots and flowers. Low phosphorus levels (LP) can promote plant growth and maintain the content of active ingredients.

### 3.2. Influences of Phosphorus Fertilization on Bupleurum Metabolites

#### 3.2.1. Effect of Phosphorus Fertilization on Sugar Metabolism of *Bupleurum*

As an indispensable energy source, sugars have a significant effect not only on plant growth, but also on biomass production. Moreover, the stress response and growth process of plants are also regulated by sugars [54]. There are two main ways for plants to respond to low phosphorus, including changes in root system architecture (RSA) and the modification of metabolic processes [55]. We found that the variation of *Bupleurum* metabolites significantly affected the accumulation of chemical components in different parts under different levels of phosphorus fertilization. The specific performance is that the content of sugars, organic acids and derivatives, polyols and derivatives and alkyl were upregulated in flowers. Furthermore, the content of these compounds in main and lateral shoots showed the same upward trend, except glycosides and polyols and derivatives. Previous studies have confirmed the increased accumulation of sugars, such as d-glucose, d-fructose and d-mannose in both main and lateral shoots [56]. The accumulation of sugars was detected in barley [57] and maize [58], in the absence of phosphorus. Therefore, sugar accumulation under low phosphorus conditions might be a phosphorus efficiency mechanism. However, Muller treated up in with phosphorus deficiency [59], and observed that glucose, fructose and sucrose in shoots, after 14 days of treatment, were reduced, while there was no significant change in the amount of sugar levels in roots and shoots with 22 days of similar treatment. As for roots, we observed that all components presented the same variation: having first increased and then decreased. Comparing CP and HP, however, the final result was up-regulated. Overall, the least accumulation of compounds was in lateral shoots and roots, which were mainly concentrated in CP and LP treatment groups. This difference can be attributed to differences in farming methods and stages of harvest. Previous results confirmed that the accumulation of sugar began in the early stage, and the accumulation speed accelerated in the mid stage until the maturity stage, and then tended to be stable [60,61]. Furthermore, different levels of phosphorus deficiency lead to different results. In fruit, sugar accumulation was largely determined by sucrose metabolism [62,63,64], indicating that the application of phosphorus fertilization can enhance fruit’s sink strength, which may increase the amount of sugar that leaves deliver to the fruit [44].

#### 3.2.2. Effect of Phosphorus Fertilization on Acid Metabolism of *Bupleurum*

Phosphoenolpyruvic acid (PEP) was closely related to the metabolic precursors of most organic acids. The effects of phosphorus deficiency on the secretion of organic acids in plant roots were as follows: short-term treatment increased the content of organic acids, while long-term phosphorus deficiency reduced the release of organic acids in the rhizosphere [51]. According to the current study, malic acid and citric acid content secreted by *Bupleurum* roots at the late stage of P deficiency increased. In plant tissues, the synthesis of organic acids was largely influenced by the citric acid cycle and glyoxylic acid cycle [56]. Comparing HP and LP, the level of malate and citrate were up-regulated in roots. However, both were down-regulated compared to the control group. Lin [65] supplied different concentrations of phosphate fertilizer on uniform tea, and the concentration of root malate and citrate did go down as the phosphorus supply increased from 0 to 40 μM, and then slightly increased with further increase the content of phosphate. The trend was same to the result we mentioned above. Additionally, the variation of content of malate and citrate showed in heat map had the same trends. Above all, we found that LP and HP treatment decreased the content of malate and citrate, which could be attributed to deceleration of TCA-cycle in roots. Inconsistent results suggest that the regulation of rhizosphere metabolism is not unique, and different phosphorus concentration may lead to contradictory results. The content of succinic acid and trans-aconitate in the rhizosphere increased when sufficient phosphorus fertilization was applied to *Z. mays* [66]. A similar reaction was found in G. max, which was manifested by an increase in the proportion of citric acid in root exudates [67], and the contents of fumaric acid and succinic acid in *Hordeum vulgare* roots increased significantly [57].

According to the content of phosphorus fertilization, a majority of research compared +P levels and -P levels to discuss the physiological and biochemical effects of phosphorus fertilizer on plants [10,44,46]. In addition, several phosphorus levels were set to discuss the optimal dosage. For example, Mohidin [68] set five phosphorus levels in combination with nitrogen and potassium to optimize oil palm seedlings in solution culture: 15 mg∙L^−1^, 30 mg∙L^−1^, 60 mg∙L^−1^, 90 mg∙L^−1^, 120 mg∙L^−1^. Additionally, according to the Dangi study [47] on the effect of phosphorus on the growth and flowering of Marigol, phosphorus levels were respectively 0 kg∙ha^−1^, 20 kg∙ha^−1^, 40 kg∙ha^−1^, 60 kg∙ha^−1^, 80 kg∙ha^−1^, 100 kg∙ha^−1^, 120 kg∙ha^−1^, and he also found that the highest plant length was obtained at 20 kg∙ha^−1^—the lowest concentration of phosphorous used. At the same time, in order to impact the phosphorus fertilizer level on the yield and metabolome of goji fruit, Feng [21] set three phosphorus levels—32.5 g∙per tree^−1^; 65 g∙per tree^−1^; and 97.5 g∙per tree^−1^—and he concluded that the yield of goji fruits had a strong negative correlation with phosphorus levels. Although these studies concluded that lower levels of phosphorous had better impact on plant yield, the lowest level used was 20 kg∙ha^−1^. In our study, we used lower levels of phosphorous 20 kg∙ha^−1^ and 10 kg∙ha^−1^, in trying to reach the optimal dosage.

## 4. Materials and Methods

### 4.1. Experimental Design and Plant Source

The medicinal plant used in our study was cultivar *Bupleurum scorzonerifolium* Willd., which was widely cultivated in the northeast of China. The dried roots of *Bupleurum chinense* DC. and *Bupleurum scorzonerifolium* Willd. were the only two authentic sources of Chaihu [69]. Two-year-old *Bupleurum* seedlings were cultivated in natural environmental conditions at the research site of Lin Dian, Da Qing, Heilongjiang province, China. The chemical prosperities of the soil [70] were pH 7.78, electrical conductivity was 134 us∙cm^−1^; and organic matter was 4.86 g∙kg^−1^. In the 20182019 growing season, the average annual temperature was 4 °C, and the average annual precipitation was 417.2 mm [71].

The three phosphorus levels were distributed in a randomized-block design, and each plot measured 3 m × 1.2 m. Based on previous experience, (Ca(H_2_PO_4_)_2_·H_2_O) was used as phosphorus fertilization. *Bupleurum* was processed for control phosphorus (CP), low phosphorus (LP) and high phosphorus (HP) treatment, by adjusting the phosphorus concentration in the current study. The three phosphorus fertilization levels were: CP (0 kg∙ha^−1^); LP (10 kg∙ha^−1^); and HP (20 kg∙ha^−1^) (Table 1). Phosphorus fertilization was applied in July 2018. After the 30th day (August 2018), the *Bupleurum* were harvested and divided into flowers, main shoots, lateral shoots and roots, and quickly transferred to the laboratory within three hours. Each group obtained three technical replicates, and the fresh weight and length were then measured. Simultaneously, each sample was divided into two batches: one batch was dried to a constant weight in a blast oven at 42 °C and used for saikosaponins extraction, and the other batch was stored at −80 °C for sample processing of GC-MS.

### 4.2. Determination of the Saikosaponins Content

**Figure 8:**
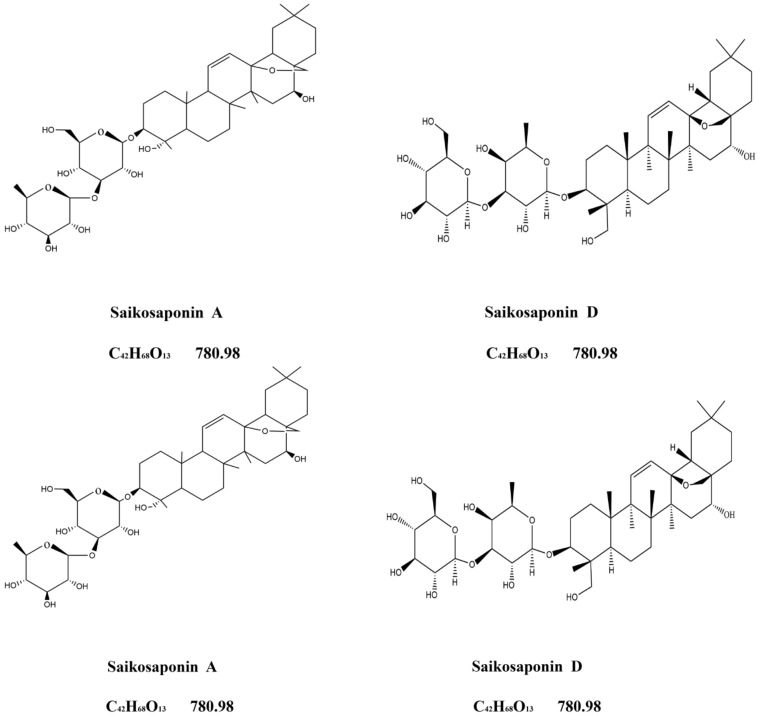
Molecular formula and molecular weight of saikosaponin A and D.

500 mg dried powder of *Bupleurum* (roots, main shoots, lateral shoots and flowers) was dissolved into 25 mL of methanol solution containing 8% ammonia water. Samples were ultrasonicated at 30 °C for 30 min, and then filtered. The filter residue was rinsed twice with 10 mL methanol, and the filtrates were combined and recovered to dryness. The residue was dissolved in methanol to a 10 mL volumetric flask. The solution was passed through a 0.45 μm filter membrane to use. An aliquot of 20 μL was injected for HPLC analysis. The content of saikosaponin A and D (Figure 8) were measured separately. A HPLC (Hitachi, Tokyo, Japan) system was equipped with L-2000 High Performance Liquid Chromatograph and L-2200 Autosampler, and a reversed phase column was adopted. According to the test requirements of saikosaponins in Chinese Pharmacopoeia, acetonitrile and pure water were used as solvents [69], and the ratio is as follows in Table 2. A Diamonsil C18 (4.6 × 250 mm, 5 μm) chromatographic column was selected, the column temperature was 25 °C, the flow rate was 0.8 mL∙min^−1^ and the detector was set at 210 nm.

### 4.3. Preparation and Extraction

60 mg various organs of *Bupleurum* (roots, main shoots, lateral shoots and flowers) were moved to a 1.5 mL centrifuge tube. Samples were extracted by 540 μL cold methanol (pre-cooled at −20 °C for use), and 60 μL of internal standard (L-2-chloro-phenylalanine, 0.3 mg∙mL^−1^, prepared in methanol) was added sequentially. After vortexing (60 Hz, 2 min) in a grinder, ultrasonic extraction was performed for 30 min. 300 μL of chloroform were added and vortexed in the grinder (20 Hz, 2 min), and then 600 μL of deionized water was again added, vortexed and extracted by ultrasonic for 30 min. At 4 °C, samples were centrifuged at 10,000 rpm for 10 min. 700 μL of supernatant was moved to a centrifuge tube, and evaporated to dryness with a rapid centrifugal concentrator. The dried residue was dissolved in 200 μL of methoxyamine pyridine solution (15 mg∙mL^−1^), and incubated in a shaking incubator at 37 °C for 90 min. Subsequently, 200 μL N,O−Bis (trimethylsilyl) trifluoroacetamide (BSTFA) and 40 μL Hexane were added, vortexed for 2 min and derivatized 60 min at 70 °C [23,25,72]. The solution was centrifuged at 12,000 rpm for 5 min, in order to obtain the supernatant for GC-MS analysis.

### 4.4. GC−MS Analysis

In this experiment, the analytical instrument was Agilent’s 7890A-5975C gas chromatography-mass spectrometer (Agilent, Santa Clara, FL, USA). 1 μL of the derivatized extract was injected into the instrument, and the sample was passed through a non-polar DB-5MS capillary. The column (30 m × 250 μm ID, J&W Scientific, Folsom, CA, USA) was separated and entered into mass spectrometry detection. High-purity helium was used as the carrier gas at a flow rate of 1.0 mL∙min^−1^. Program temperature [23,25,72]: 8 °C∙min^−1^, from 0 °C to125 °C; 4 °C∙min^−1^, from 125 to 210 °C; 5 °C∙min^−1^, from 210 to 270 °C; 10 °C∙min^−1^, from 270 to 305 °C; and a final maintenance at 305 °C for 3 min. The temperature of the injection port and EI source were both 260 °C, and the voltage was -70 V. The mass scanning range was *m*/*z* 50–600, and the acquisition rate was 20 spectrum∙s^−1^.

### 4.5. Multivariate Statistical Analysis

The partial least squares discriminant analysis (PLS-DA) method was used to comprehensively compare the identified metabolites. According to the definition of fold change being less than 0.05 and *p* value being greater than 1 in Student’s t-test analysis, the differential metabolites were selected. The Kyoto Genome and Genome Encyclopedia (KEGG) were used to analyze the pathways of metabolites. The difference in the biomass and metabolite among CP, LP and HP were calculated using a one-way analysis of variance. The Graph Pad Prism 9.0 was used for drawing related graphs in article. Using hierarchical cluster analysis, the content value of each compound was standardized to obtain a heat map of relative differences.

## 5. Conclusions

The findings of the current research highlighted the effect of phosphorus fertilizer on the traditional Chinese medicinal plant *Bupleurum.* By comparing the indexes and metabolomics analysis results of *Bupleurum* under three phosphorus fertilizer levels, it can be concluded that phosphorus plays a pivotal role in the growth of *Bupleurum*. The application of high level of phosphorus could not significantly prompt the increase in fresh weight, length and saikosaponins active metabolites, but enriched the plants with primary nutrients. Thus, we can say that although phosphorus is essential for plant growth, high phosphorus level is not preferable for maintaining the yield and quality of *Bupleurum.* Meanwhile, a proper amount of phosphorus could promote the yield and quality characteristics, especially in the above-ground parts of *Bupleurum*. Moreover, metabolomics analysis identified 73 kinds of metabolites of *Bupleurum* under phosphorus stress (CP, LP and HP). Under high phosphorus levels, the content of sugars, organic acids and derivatives, polyols and derivatives and alkyl were upregulated in flowers. Additionally, the contents of compound in main shoot and lateral shoots showed the same upward trend, except for glycosides, polyols and derivatives. In addition, 4 differentially accumulated metabolites in *Bupleurum*—ether, glycerol, chlorogenic and l-rhamnose—were identified as key metabolites under phosphorus fertilization. The identified metabolic pathways were mostly starch and sucrose metabolism and galactose metabolism, which set up a primary metabolic network diagram associated with *Bupleurum*. Above all, the impact of phosphorus fertilizer on *Bupleurum* is obvious, and it is meaningful for us to further explore the influence of phosphorus fertilizer on related Traditional Chinese Medicinal plants.

## Figures and Tables

**Table 1:** Amount of phosphorus fertilization applied to *Bupleurum*.

Treatment	CP	LP	HP
Phosphorus fertilization	0 kg∙ha^−1^	10 kg∙ha^−1^	20 kg∙ha^−1^

**Table 2:** Elution gradient.

Times (min)	Acetonitrile (%)	Pure Water (%)
0–50	25–90%	75–10%
50–55	90%	10%

## Data Availability

Not applicable.
